# Supplementary material for: Information overload and parental perspectives on information provided to parents/carers of paediatric patients undergoing elective surgical procedures
Source: PLoS One. 2024 Oct 22;19(10):e0309485. doi: 10.1371/journal.pone.0309485 (PMC11495572; doi:10.1371/journal.pone.0309485)
Supplement: S1 File — (PDF) [file pone.0309485.s001.pdf]

POLARBEAR Interview guide

Thank you for agreeing to participate in our POLARBEAR study. We are a group of researchers from Perth Children's Hospital, The University of Western Australia and the Telethon Kids Institute working on a study assessing whether the level of information given to families of children undergoing elective surgery is appropriate. The purpose of this interview is to explore, from the parents' perspectives, ways to make sure the right amount of information is provided to all families without overloading our families with information.

Ask parent to complete the BRIEF Health Literacy tool and demographic data sheet and to re-confirm consent for the study.

Now I'm going to ask you a number of questions relating to how you personally feel about the information you received when your child had elective surgery at PCH. There are no right or wrong answers, and you do not need to answer any questions you don't feel comfortable answering. We want to know your thoughts and we value your opinion. Did you have any questions before we begin?

1. Ask the participant about their experiences with elective surgery for their children.

2. How do you feel about the variety of recommendations about caring for your child after surgery?
   - PROMPT: By recommendations, we mean information that you may have been told or written information you may have been given by someone, as well as information that you may come across yourself, e.g. by looking online.
   - FOLLOW-UP: Where did these recommendations come from? Surgeons? Nurses? Anaesthetists? GP? Family members? Friends? Online/social media?
   - FOLLOW-UP: how did you keep track of the recommendations?
   - FOLLOW-UP: How did you know which ones to follow?
   - FOLLOW-UP: Which ones do you think were the most trustworthy?
     - FOLLOW-UP -up: why is that?
   - FOLLOW-UP: If participant talks about feeling overwhelmed with the number of recommendations/complexity of recommendations
     - FOLLOW-UP: What do you do when you feel overwhelmed with the recommendations?

3. Was there anything about how the information was phrased that made it confusing?
   - PROMPT: What parts of the information made it difficult for you to understand what they are trying to say?

- FOLLOW-UP: Why is that?

4. What could be done to help you take on and make full use of the information you are being given when your child is undergoing elective surgery?
    - PROMPT: would you like to receive the information in a particular format e.g.?
    - PROMPT: what advice would you give a friend who had a child undergoing elective surgery?

At interview conclusion:
Thank you for your time today, you've provided a lot of comments. What other comments did you have?

**Field Notes**
Participant Name: ____
Study number P________
Researcher name: ____ Date: ___________
