# Supplementary material for: Information overload and parental perspectives on information provided to parents/carers of paediatric patients undergoing elective surgical procedures
Source: PLoS One. 2024 Oct 22;19(10):e0309485. doi: 10.1371/journal.pone.0309485 (PMC11495572; doi:10.1371/journal.pone.0309485)
Supplement: S2 File — (PDF) [file pone.0309485.s002.pdf]

SUPPLEMENT 2 for Information overload and parental perspectives on information provided to parents/carers of paediatric patients undergoing elective surgical procedures.

For the correlation matrix for the five-item CIO score, the KMO value was 0.78, and the Bartlett test of sphericity was significant at 5% ($\chi^2(10)$=526.8, p<0.001). These values indicate that the data were suitable for factor analysis. Cronbach's alpha was 0.78, indicating acceptable internal consistency.

A Scree plot for EFA-determined eigenvalues indicates a one- or two-factor solution, while parallel analaysis indicated a 2-factor solution (S2 Fig 1). This suggests that the modified 5-item CIO scale for postoperative information overload measures two latent construct. Using Principal Axis Factoring with no rotation, all five items loaded onto one factor, with 47% of the total variance explained by this factor.

S2 Figure 1. Scree plot (left) and parallel analysis (right) for the correlation matrix of the five items in the modified CIO score.

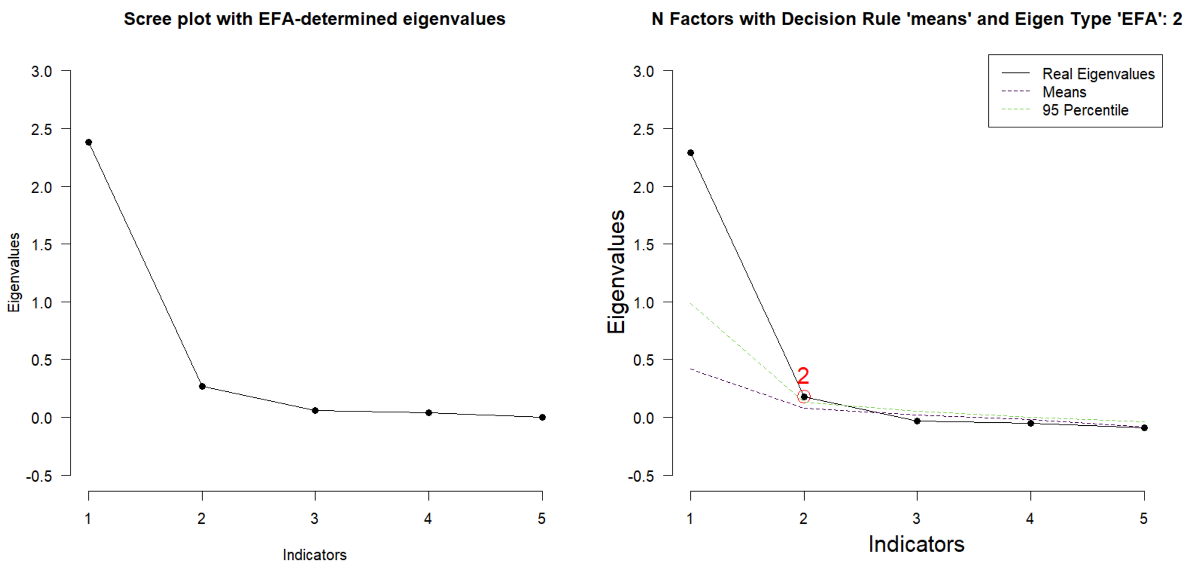

S2 Table 1. Factor loadings of 5-item modified CIO score items on two-factor solution.

	Factor 1	Factor 2
Eigenvalue	2.36	0.24
Proportion of Variance	0.47	0.05
Cumulative Proportion of Variance	0.47	0.52
**Item loadings**		
Item 1	0.585	0.216
Item 2	0.626	0.182
Item 3	0.560	0.232
Item 4	0.804	-0.238
Item 5	0.814	-0.220
