# Supplementary material for: Information overload and parental perspectives on information provided to parents/carers of paediatric patients undergoing elective surgical procedures
Source: PLoS One. 2024 Oct 22;19(10):e0309485. doi: 10.1371/journal.pone.0309485 (PMC11495572; doi:10.1371/journal.pone.0309485)
Supplement: S3 File — (PDF) [file pone.0309485.s003.pdf]

SUPPLEMENT 3 for Information overload and parental perspectives on information provided to parents/carers of paediatric patients undergoing elective surgical procedures.

S3 Fig 1. Mean and bootstrapped 95% CI of CIO scores for participants grouped by education and Aboriginal heritage.

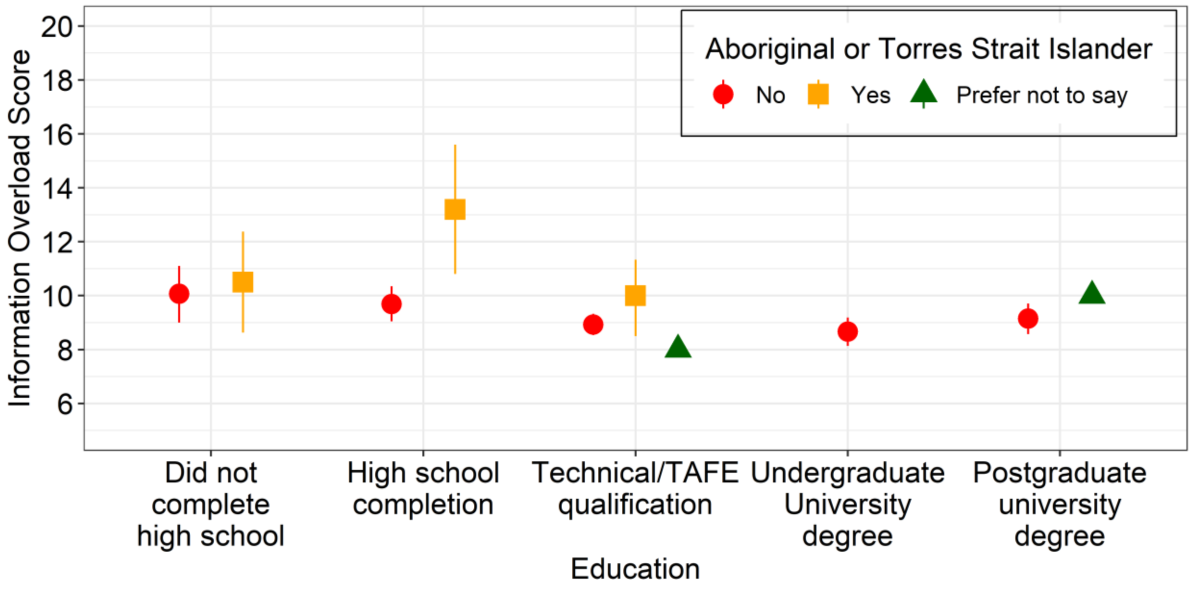

S3 Fig 2. Mean and bootstrapped 95% CI of CIO scores for participants grouped by education and age group.

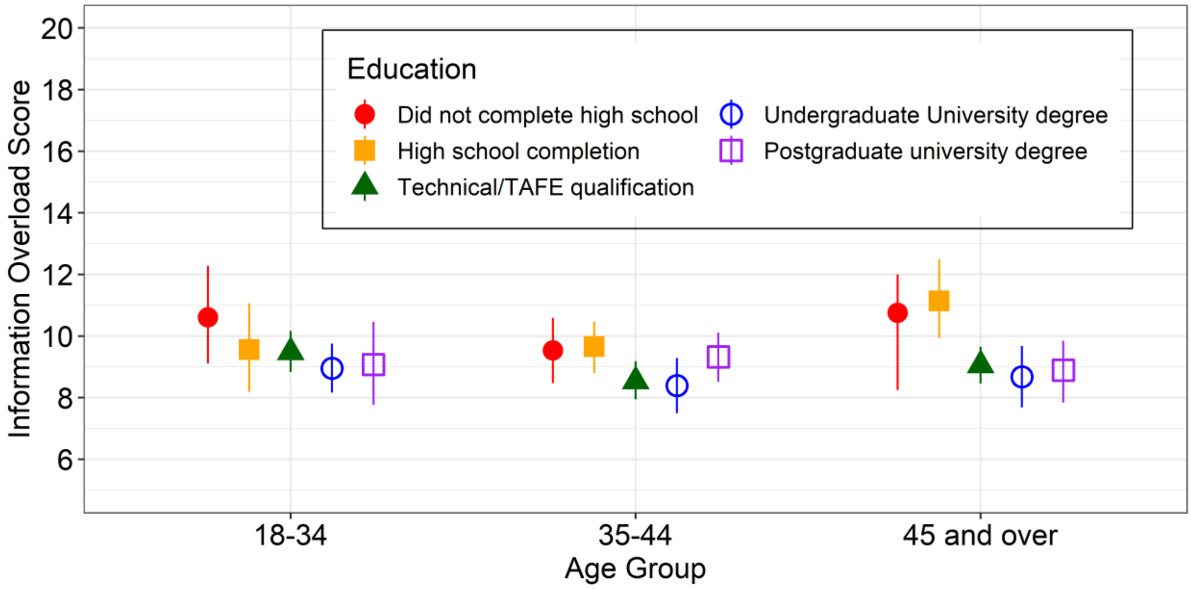

S3 Fig 3. Mean and bootstrapped 95% CI of CIO scores for participants grouped by education and whether they speak a language other than English at home.

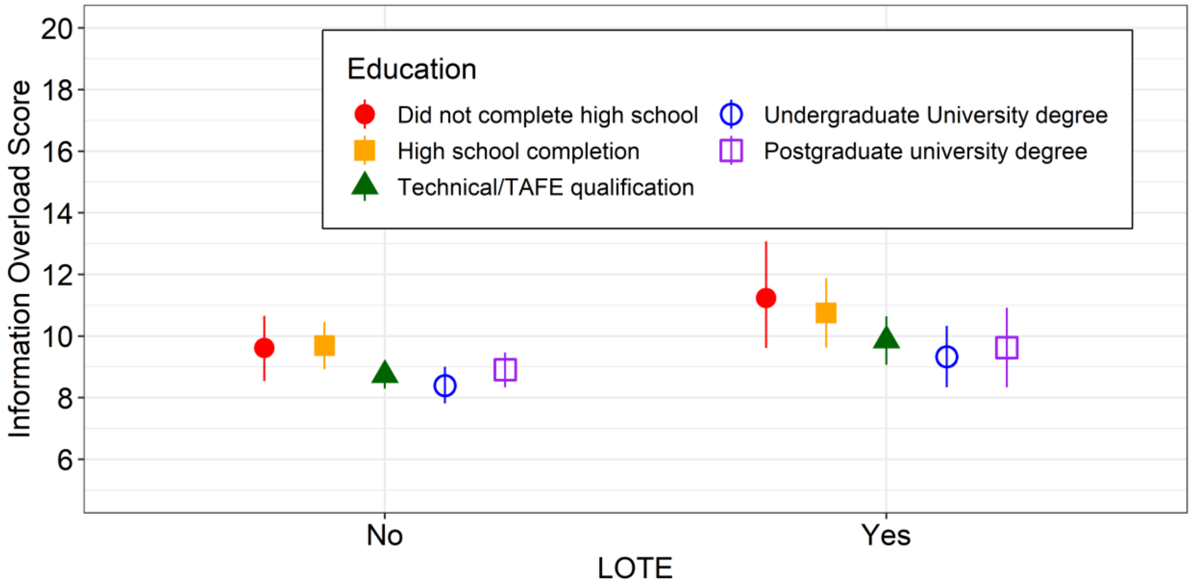

S3 Table 1. Parameter estimates from individual single-variable regression models for total score on each candidate independent variable.

Variable		Estimate	Std. Error	p-value	95% C.I.	
					2.5%	97.5%
**Aboriginal or Torres Strait Islander**	No	Ref	NA	NA	NA	NA
	Yes	1.92	0.58	<0.001	0.79	3.05
	Prefer not to say	-0.13	1.73	0.94	-3.53	3.27
**Age group (years)**	18-34	Ref	NA	NA	NA	NA
	35-44	-0.48	0.3	0.11	-1.07	0.11
	45 and over	-0.26	0.35	0.45	-0.95	0.42
**Education**	Did not complete high school	Ref	NA	NA	NA	NA
	High school completion	-0.31	0.5	0.53	-1.28	0.66
	Technical/TAFE qualification	-1.31	0.45	<0.001	-2.19	-0.43
	Undergraduate University degree	-1.67	0.49	<0.001	-2.63	-0.72
	Postgraduate university degree	-1.1	0.49	0.02	-2.06	-0.14
**Employment status**	Not currently employed, not looking for a job	Ref	NA	NA	NA	NA
	Not currently employed, but searching for a job	0.01	0.67	0.99	-1.3	1.32
	Employed, part time hours	-0.51	0.39	0.19	-1.27	0.25
	Employed, full time hours	-0.42	0.39	0.28	-1.19	0.35
	Self-employed/business owner	-0.5	0.47	0.29	-1.43	0.42
**Gender**	Male	Ref	NA	NA	NA	NA
	Female	-0.52	0.3	0.09	-1.12	0.07

**Income (per year)**	Less than $25,000 per year	Ref	NA	NA	NA	NA
	$25, 000 to $75, 000 per year	-0.69	0.59	0.24	-1.85	0.47
	$76, 000 to $125, 000 per year	-1.3	0.59	0.03	-2.46	-0.14
	$126, 000 -$250,000 per year	-1.72	0.59	<0.001	-2.87	-0.56
	Over $ 250,000 per year	-1.41	0.72	0.05	-2.83	0
	Prefer not to say	-0.69	0.64	0.28	-1.94	0.55
**LOTE**	No	Ref	NA	NA	NA	NA
	Yes	1.06	0.28	<0.001	0.5	1.61
**Home postcode**	Metro	Ref	NA	NA	NA	NA
	Regional	-0.34	0.36	0.35	-1.05	0.37
	Remote	-0.22	0.64	0.73	-1.47	1.02
**Previous surgery**	No	Ref	NA	NA	NA	NA
	Yes	-0.13	0.26	0.6	-0.64	0.37

S3 Table 2. Parameter estimates from the model for total score before backward stepwise reduction of covariates.

Variable		Estimate	Std. Error	p-value	95% C.I.	
					2.5%	97.5%
**Intercept**		11.41	0.9	<0.001	9.65	13.17
**Aboriginal or Torres Strait Islander**	No	REF				
	Yes	-0.21	1.04	0.839	-2.26	1.84
	Prefer not to say	0.42	2.59	0.87	-4.67	5.51
**Age group (years)**	18-34	REF				
	35-44	-1.08	0.84	0.203	-2.73	0.58
	45 and over	-0.68	1.36	0.617	-3.35	1.99
**Education**	Did not complete high school	REF				
	High school completion	-1.01	0.91	0.268	-2.81	0.78
	Technical/TAFE qualification	-1.19	0.8	0.136	-2.75	0.38
	Undergraduate University degree	-1.8	0.88	**0.042**	-3.54	-0.06
	Postgraduate university degree	-1.4	1	0.162	-3.37	0.57
**Employment status**	Not currently employed, not searching for a job	REF				
	Not currently employed, but searching for a job	-0.46	0.67	0.494	-1.78	0.86
	Employed, part-time hours	-0.01	0.41	0.988	-0.81	0.8
	Employed, full-time hours	0.2	0.45	0.65	-0.68	1.08
	Self-employed/business owner	-0.24	0.49	0.623	-1.22	0.73
**Gender**	Male	REF				
	Female	-0.42	0.35	0.226	-1.11	0.26
	Less than $25,000	REF				

**Income (per year)**	$25,000 to $75,000	-0.49	0.64	0.442	-1.74	0.76
	$76,000 to $125,000	-0.92	0.65	0.158	-2.19	0.36
	$126,000 to $250,000	-1.08	0.65	0.097	-2.37	0.2
	Over $250,000	-0.85	0.79	0.284	-2.41	0.71
	Prefer not to say	-0.52	0.68	0.441	-1.86	0.81
**LOTE**	No	REF				
	Yes	1.37	0.89	0.126	-0.39	3.12
**Home postcode**	Metro	REF				
	Regional	-0.24	0.38	0.531	-0.99	0.51
	Remote	-0.72	0.65	0.27	-2	0.56
**Previous surgery**	No	REF				
	Yes	0.07	0.27	0.8	-0.46	0.6
**Aboriginal status * Education**	Yes * High school completion	3.52	1.55	**0.024**	0.47	6.56
	Yes * Technical/TAFE qualification	0.74	1.48	0.618	-2.17	3.66
	Prefer not to say * Technical/TAFE qualification	-1.17	3.57	0.743	-8.18	5.84
**Age group * Education**	35-44 * High school completion	1.23	1.12	0.272	-0.97	3.43
	45 and over * High school completion	2.07	1.66	0.213	-1.19	5.33
	35-44 * Technical/TAFE qualification	0.41	0.98	0.674	-1.52	2.35
	45 and over * Techincal/TAFE qualification	0.39	1.47	0.791	-2.51	3.29
	35-44 * Undergraduate university degree	0.86	1.09	0.429	-1.28	3.01
	45 and over * Undergraduate university degree	0.48	1.55	0.756	-2.56	3.52
	35-44 * Postgraduate university degree	1.33	1.18	0.259	-0.98	3.64

	45 and over * Postgraduate university degree	0.56	1.63	0.732	-2.65	3.76
**Education * LOTE**	High school completion * Yes	-1.09	1.16	0.346	-3.37	1.18
	Techinical/TAFE qualification * Yes	-0.6	1.05	0.569	-2.67	1.47
	Undergraduate university degree * Yes	-0.57	1.09	0.604	-2.72	1.58
	Postgraduate university degree * Yes	-0.91	1.08	0.4	-3.04	1.22
