# Supplementary material for: Information overload and parental perspectives on information provided to parents/carers of paediatric patients undergoing elective surgical procedures
Source: PLoS One. 2024 Oct 22;19(10):e0309485. doi: 10.1371/journal.pone.0309485 (PMC11495572; doi:10.1371/journal.pone.0309485)
Supplement: S4 File — (PDF) [file pone.0309485.s004.pdf]

SUPPLEMENT 4 for Information overload and parental perspectives on information provided to parents/carers of paediatric patients undergoing elective surgical procedures.

The questions presented to participants in the Day-of-Surgery Survey are listed below. These questions were administered in addition to the modified 5-item CIO questionnaire. The survey was administered online via Qualtrics.

1. Which age group (in years) do you fit into?
    a. 18-24
    b. 25-34
    c. 35-44
    d. 45-54
    e. 55 and over
2. Which gender do you identify with?
    a. Male
    b. Female
    c. Non-binary gender
    d. Prefer not to say
3. Do you identify as Aboriginal or Torres Strait Islander?
    a. Yes
    b. No
    c. Prefer not to say
4. Is the main language spoken at home a language OTHER than English?
    a. Yes
    b. No
5. What is your residential postcode? ___________

6. Which category does you annual houeshold income fall into?
    a. Less than $25,000 per year
    b. $25,000 to $75,000 per year
    c. $76,000 to $125,000 per year
    d. $126,000 to $250,000 per year
    e. Over $250,000 per year
    f. Prefer not to say
7. What best describes your highest education level achieved?
    a. Did not complete high school
    b. High school completion
    c. Technical/TAFE qualification
    d. Undergraduate university degree
    e. Postgraduate university degree
8. Which of the following best describes your employment status?
    a. Not currently employed, not looking for a job
    b. Not currently employed, but searching for a job
    c. Employed, part time hours
    d. Employed, full time hours
    e. Self-employed/business owner
9. Have you or any of your children had a similar surgery previously?
    a. Yes
    b. No
10. Have you or any of your children had multiple surgeries previously?
    a. Yes
    b. No

11. How many health professionals has your child seen prior to this surgery in relation to the surgery/condition? This includes doctors, GPs, surgeons, nurses, pharmacists, physiotherapists, OT, dentists etc
    a. One
    b. Two – five
    c. More than five
