# Supplementary material for: Information overload and parental perspectives on information provided to parents/carers of paediatric patients undergoing elective surgical procedures
Source: PLoS One. 2024 Oct 22;19(10):e0309485. doi: 10.1371/journal.pone.0309485 (PMC11495572; doi:10.1371/journal.pone.0309485)
Supplement: Attachment — Submitted filename: Response to Reviewers.docx [file pone.0309485.s005.docx]

Below are our responses to the points raised by the reviewers.

**REVIEWER #1:** 
**Is the manuscript presented in an intelligible fashion and written in standard English?
No. However, the authors are clearly proficient in English, so “No” is related to the reporting of statistical analyses and results. The manuscript would profit from a careful reading by a statistician or others with similar competencies. The STROBE reporting guidelines may also be helpful in this respect.**

**Authors’ response:** One of the authors is an experienced statistician Dr Nazim Khan who has clarified sections in the revised manuscript.

**Review comments to the Authors
The paper investigates information overload as reported by parents or carers of pediatric patients undergoing elective surgical procedures. A strength of the study is that both quantitative (survey and interview) and qualitative (interview) data are collected. The study may be perceived as a mixed methods (MM) study. If it was planned as such, and depending on the chosen MM design, all parts of the paper will need major revision. If not, there are still other issues that warrant major revisions. These are addressed below.**

**Authors’ response:** We have addressed the issues raised in the revised manuscript.

**Introduction
The authors may consider using a more comprehensive definition of health literacy, possibly in line with Sørensen et al. (2012), DOI: 10.1186/1471-2458-12-80.**

**Authors’ response**: The definition has been updated in the revision.

**The research gap should be stated clearer. It is not explicitly communicated whether research exist that would answer the research question.**

**Authors’ response:** This has now been explicitly stated.

**Some statements in the last paragraph miss references. This concerns the sentences starting with “Parents attending …..”, and “Furthermore, the perioperative time ….” .**

**Authors’ response:** References have been added.

**The introduction should give the rationale for collecting both quantitative and qualitative data. Are the two approaches meant to give complementary data, or are there other reasons? E.g. on page 5, the survey is denoted as “stage 1” indicating a sequential design.**

**Authors’ response:** This has now been clarified in the revised manuscript. The quantitative and qualitative information is meant to give complementary data. First collecting overarching data and then diving deeper in the issues.

**STROBE recommend that objectives are stated as part of the introduction.**

**Authors’ response:** This has now been clarified in the introduction.

**Materials and methods
Objectives
I cannot see that the paper research and reports on the utility of the scale (objective 1).**

**Authors’ response:** This had now been further clarified in the revised manuscript.

**Although it may be considered as a separate research question, objective 2 could be expanded to: …identify the level and explore factors associated with …**

**Authors’ response:** This is an interesting idea that could be pursued in future research.

**Survey; material and analyses
Information should be given as to when and where parents/carers were approached, e.g. where the poster was located.**

**Authors’ response:** This has now been clarified in the revised manuscript.

**Any categorizations of variables (like for post code) should be described.**

**Authors’ response:** Thank you for pointing out this omission, we have added further detail as to the classification of postcodes into metropolitan (metro) or rural status to our methods section as follows: “Postcodes were classified as metropolitan (metro), regional, or remote based on the Australian Bureau of Statistics Statistical Geography Standard Correspondences Postcode 2017 to Remoteness Area 2016. Postcodes classified as being in “Major Cities of Australia” were grouped under “metro”, postcodes classified as “Inner Regional Australia” or “Outer Regional Australia” were grouped under “regional”, and those classified as “Remote Australia” or “Very Remote Australia” were grouped under “remote”.”

**If a statistical power analysis was performed before study start the result should be reported.**

**Authors’ response:** Given that there is no previous data to base our calculations on, we were unable to perform a power calculation. Further, as a key objective of the quantitative component of our study was to estimate prevalence of information overload, we used conservative parameters to estimation sample size based on population proportion. We have updated our manuscript to clarify this.

**How the regression model-building was performed is not clear. 1) Regarding the inclusion of interaction terms, the phrase “…as appropriate based on data exploration.”, needs to be elaborated. 2) It is unclear whether interaction terms were included or not in the stepwise regression analysis. Note that “removing stepwise” is not commonly used to describe a backward stepwise regression.**

**Authors’ response:** The Data Analysis methods have been revised for clarity. Additionally, relevant interaction plots have been included as supplementary figures. The model-building methodology now reads: “Variables considered in the regression analysis included participants’ demographic characteristics (gender, age group, Aboriginal heritage, whether a language other than English is spoken at home, education level, employment status, income, postcode remoteness) and whether or not the respondent or any of their children had previously had a similar surgery. Interaction terms between variables were included based on exploration of interaction plots and cross-tables. Backwards stepwise regression was used to reduce the model to significant covariates, where statistical significance was taken at p<0.05.”

**In the discussion (lines 324-327) the authors write that low education level, low health literacy, poor searching skills, greater concern about information quality, low socioeconomic status, and race (Hispanic and non-Hispanic black) are positively associated with information overload with reference to Khaleel et al. (2020). In methods (page 8) only education is highlighted. Why?**

**Authors’ response:** We did state that the interaction between Aboriginal status and education is significant, and this relates to the finding by Khaleel et al. regarding race. However, in our data we did not find a correlation with annual household income and employment status, which are proxies for socioeconomic status. We have made this comparison with Khaleel et al. more explicit in the discussion.

**Information should be given on when the interviews were performed (time since the operation).**

**Authors’ response:** The interviews were all conducted between 20 and 59 days following the operation. A statement on this has been added to the manuscript.

**The authors may consider providing the interview guide as an additional file.**

**Authors’ response:** The ethics committee approved Interview Guide has been included as suggested.

**Lines 150 (The demographic …) – 151: It is unclear whether demographic data was collected during the interview or was retrieved from existing survey data.**

**Authors’ response:** The demographic data was collected during the interview. A sentence has been added to clarify this.

**Lines 152 (Thematic ….) - 153 is a description of analyses and should be moved to the analysis section (or removed if already given).**

**Authors’ response:** This has been moved to analysis.

**It should be stated explicitly what phenomena barriers and facilitators points to, either in methods or results – whatever is the more appropriate.**

**Authors’ response:** The significance is addressed in the discussion section.

**Lines 191 -194 (until A total of…) describes methods and should be moved or deleted.**

**Authors’ response:** Thank you, these have been removed as they are a repeat of what is already in the methods.

**Is it important that it is 15 %? Does the number indicate a rule for inclusion of reported data?**

**Authors’ response**: It is not important that is less than 15% particularly, we have re-phrased this sentence for clarity. It now reads: “Of these, 2 participants did not respond to any of the survey questions following consent, and were subsequently excluded from analysis…”

**Mean and standard deviation of the sum score should be provided.**

**Authors’ response:** For consistency, the median (LQ, UQ) of the sum score has been reported as well as the mean and standard deviation. The median (LQ, UQ) of the total score has been added to Table 3, and the mean, SD, minimum and maximum scores reported in the text.

**The phrase “insignificant covariates” should be used with caution. “Not statistically significant” would be more appropriate.**

**Authors’ response:**  The relevant sentence has been amended in the text, and now reads: “Following backwards stepwise regression, the estimated coefficients…”

**Results from simple (unadjusted) regression analyses should be considered presented in an additional file. This will increase the transparency of the analyses, in particular as a stepwise regression procedure was used.**

**Authors’ response:** We assume the reviewer is referring to the full model before covariate reduction, given that single-variable regression models are generally misleading since they don’t consider interactions and effects of covariates. To address this, the table of estimates from the model before covariate reduction has been included as an appendix to the manuscript.

**It is unclear whether all results in table 4 comes from a regression model including interaction terms between aboriginal status and education. If that is the case, the interpretation of the estimated main effects is incorrect.**

**Authors’ response**: The manuscript text under Results – Regression analysis has been amended for clarity based on this feedback. The discussion has also been appropriately amended.

**Use of the reporting guideline (COREQ) should be stated under methods.**

**Authors’ response:** A statement on the use of COREQ has been added to the methods section.

**Lines 253 (Willing parents…) - 254 may be considered moved to methods.**

**Authors’ response:** this sentence moved to methods section as suggested.

**A detail, but both the terms facilitators and enablers are used.**

**Authors’ response:** We have changed *faciltators* in the table to *enablers* which to ensure that only the term enabler is used throughout the manuscript

**If barriers, facilitators and overloaded were the main themes identified this should be stated explicitly. It is now not clear whether these were identified or pre-defined.**

**Authors’ response:** These were identified and this has been clarified in the revised manuscript.

**Subheadings (barriers etc.) may increase the readability of this section.**

**Authors’ response:** We respectfully disagree since it would take away the flow of the discussion.

**The first paragraph should give a much shorter and less detailed overview of the results**

**Authors’ response:** This section has been revised.

**Strengths: Suggest more explicit statements of what was gained by retrieving both quantitative and qualitative data.**

**Authors’ response:** This has been clarified in the revised manuscript.

**It is** **a limitation of the study that information on health literacy was not collected in the survey.**

**Authors’ response:** A statement to this affect has been added to the Limitation section at the end of the manuscript.

**Reviewer #2:** 
**The transformation of the CIO measurement by simply removing the cancer word is not usually recommended. Authors should report the validity and the reliability of the new scale and whether the new scale has been pilot tested.**

**Authors’ response:** We followed the methodology used in the article of Obamiro K, Lee K. (Information overload in patients with atrial fibrillation: Can the cancer information overload (CIO) scale be used? Patient Educ Couns. 2019;102(3):550-4).We never intended to validate the scale.

**It would be helpful to know what types of surgeries the children are undergoing and other demographic characteristics and their primary diagnosis when interpreting the results. The null findings is likely due to the non-cancer related diagnosis.**

**Authors’ response:** We agree that it would have been ideal to consider further characteristics and surgery type in the analysis, however we did not collect this information from participants. All participant characteristic information that was collected has been included in the analysis. The word cancer, in line with the article from Obamiro K, Lee K. (2019), was removed from the scale and replaced with caring for your child after surgery. Therefore, the type of surgery was not collected as we were looking for an overall impression of parents experience regardless of surgery type and diagnosis.
