# Supplementary material for: Information overload and parental perspectives on information provided to parents/carers of paediatric patients undergoing elective surgical procedures
Source: PLoS One. 2024 Oct 22;19(10):e0309485. doi: 10.1371/journal.pone.0309485 (PMC11495572; doi:10.1371/journal.pone.0309485)
Supplement: Attachment — Submitted filename: Response to Reviewers.docx [file pone.0309485.s007.docx]

Below are our responses to the points raised by the reviewers.

**REVIEWER #1:**

**I agree that unadjusted estimates may be misleading due to observed and unobserved confounders (and of course other modelling issues). However, the reason for providing both are as is pointed out in the STROBE guideline, 16a: “Readers can compare unadjusted measures of association with those adjusted for potential confounders and judge by how much, and in what direction, they changed.” S2 table 1 provides estimates from the full model, including interaction terms, before the backward stepwise procedure, and so does not give unadjusted (crude) estimates. My conclusion was therefore that the statistical analysis had not been performed appropriately and rigorously. This may however be an issue best decided by the editor.**

**Authors’ Response:** We have now added a table of the unadjusted single-variable regression estimates to Supplement 2.

**The authors write in their response:” When interaction terms are used in statistical modelling, care must be taken not to interpret the main effects of each variable in isolation”. This was exactly my point, so thanks for the clarification.**

**Authors’ Response:** We thank the reviewer for their comments on the clarity of the statistical modelling, which allowed us to improve the communication of the regression modelling results.

**REVIEWER #2:**

**Thank you for your important work on addressing parents experiences particularly information overload in healthcare settings.**

**Authors’ Response:** We thank the reviewer for their comments which led to the improvement of the quality of our manuscript.

**REVIEWER #3:**

**I have been invited to review this interesting paper which has previously been reviewed twice by others. I agree that Information Overload is a potentially important issue for parents caring for children undergoing surgery.**

**I have some concerns about the data collected, the reporting of the methods used for data collection and analyses, the reporting of the analyses (with a focus on the qualitative analysis) and the interpretation of the results which has led me to answer “No” to most of the questions. I answered Don't Know to the Statistical Analysis question as I have not been able to allocate sufficient time to review the statistical analysis in sufficient depth to offer a fair evaluation. Below is a summary of my concerns and suggestions from revisions.**

**Authors’ Response:** Thank you for your comments, our answers are presented one by one below.

**Adaption of the Cancer Information Overload measure**

**The wording in your adapted measure appears to be significantly different from the original version, beyond a simple switch of the word “cancer” for “caring for your children after surgery” as stated in line 141. This leads me to have concerns about whether the modified scale is measuring information overload as intended or could potentially be measuring something else. There doesn’t appear to be a report of any pre-piloting to provide confirmation that the items are understand as intended by parents.**

**For example:**

**Item 2 (original) “There is so much cancer information, I don't even care to hear new things about cancer”.**

**Item 2 (modified) “It has gotten to the point where I don’t even care to hear new information about caring for my child after surgery”.**

**Authors’ Response:** We apologise that we were unclear about the origins of the scale used in our study. It is based on the modified 5 item Cancer Information Overload Scale (Costa et al) and an 8-item atrial fibrillation information overload scale published by one of the authors on this manuscript (KL). The table below shows how these three scales compare:

| **Atrial fibrillation scale (Obarimo and Lee 2019)** | **Modified CIO scale (Costa et al 2015.** | **Scale used by us** |
| --- | --- | --- |
| There are so many different recommendations about managing atrial fibrillation, it’s hard to know which ones to follow | There are so many different recommendations about cancer, it’s hard to know which ones to follow. | There are so many different recommendations about caring for your child after surgery, it’s hard to know which ones to follow |
| There is not enough time to do all of the things recommended to manage atrial fibrillation |  | N/A |
| It has gotten to the point where I don’t even care to hear new information about atrial fibrillation | There is so much cancer information, I don't even care to hear new things about cancer. | It has gotten to the point where I don’t even care to hear new information about caring for my child after surgery |
| No one could actually do all of the atrial fibrillation management recommendations that are given |  | N/A |
| Information about atrial fibrillation all starts to sound the same after a while | There is so much information about cancer, it all starts to sound the same after a while. | Information about caring for my child after surgery all starts to sound the same after a while |
| I forget most of the information about atrial fibrillation right after I hear it | There is so much cancer information, I forget most cancer information right after I learn it. | I forget most of the information about caring for my child after surgery right after I hear it |
| Most things I hear or read about atrial fibrillation seem pretty far-fetched |  | N/A |
| I feel overloaded by the amount of information about atrial fibrillation I am supposed to know | I feel overloaded by the amount of cancer information I am supposed to know. | I feel overloaded by the amount of information about caring for my child after surgery I am supposed to know |

We have made changes to the manuscript to make this clearer and called our scale an information overload scale throughout. Please note that we while we didn’t pre-audit the scale used, we did consult with our Anaesthesia Research Consumer Reference Panel which consists of a diverse group of parents/consumers, and they were happy with the scale.

**It would also be helpful to have a statement about the readability of the modified items to ensure that the items are comprehensible to parents of varying educational attainment levels. It would be possible to test readability retrospectively.**

**Authors’ Response:** We work very closely with consumers in all our projects. Our consumer research reference panel is a panel with members of diverse backgrounds, including CALD members and indigenous members. All questions were designed with the help of our consumer partners to ensure readability for a wide range of parents. The

**The EFA was used to explore the factor structure of the scale. It would be helpful to have a sentence in the methods or results that links the EFA to the decision to sum the 5 items to form a one factor scale.**

**Authors’ Response:** The score was summed to align with the original information overload score and previously published adjusted CIO scores. A sentence has been added to the results to reflect this:

*Exploration of the factor structure indicated a one- or two-factor solution; however, the individual modified information overload scale items were summed to a one-factor solution to align with the design of the original CIO score.*

**Qualitative study**

**More information is needed to enable the reader to evaluate the qualitative study data. It would be helpful to have a brief reference to the authors’ epistemological stance along with a statement of reflexivity which is helpful to understand the relationship of the researchers to the data collection and insight generation process.**

**Authors’ Response:** We have added in the following paragraph “Analyst triangulation was used during the coding process to improve the reliability of the data, acknowledging that each researcher brings their own attitudes, values and world views to their interpretation of the interview transcripts. Incorporating multiple researcher viewpoints and skillsets into the analysis mitigates the risk of bias or favoring a particular point of view. Prior to analysis, each researcher also reflected on their pre-existing perspectives, assumptions and expectations of the data in order to identify implicit biases, thus further improving the reliability of the data.”

**More information is needed about the sampling strategy. 35.6% (n=136) agreed to be interviewed, it would be good to know what the strategy was to decide who to contact – was a purposive or consecutive sampling approach taken and with what justification.**

**Authors’ Response:** Recruitment was consecutive convenience sampling based on staff availability. Families were invited whenever research staff was available for recruitment. When staff was available, all potentially eligible families were contacted.

**It would be great to have a more detailed description of the analytical process undertaken to develop the coding framework – including reference to whether those who completed the analysis were the same or different people to those who conducted the interviews, was coding inductive or deductive, how did you arrive at the key themes of ‘barriers’ and ‘facilitators’ and what did the barriers and facilitators relate to – information overload or general communication or something else?**

**Authors’ Response:** Additional information has been added to the manuscript to describe the process.

**Presentation of quotes from participants should ideally be at a minimum labelled with a participant ID so that the reader can be reassured that the quote come from a number of participants. It can also be useful to have other descriptives e.g. gender, or education level so that there is some contextual information to help interpret the quotes offered.**

**Authors’ Response:** We have added in a “number” column for each subcode to ensure that readers are aware that subcodes were discussed by multiple participants. We have included the participant ID after each quote. Quotes are included to exemplify the key themes identified, rather then explore an individual patients perspective. We therefore believe descriptive or contextual information for each quote is not required. General demographic information is included for those who completed the qualitative interview stage.

**Relationship between the quantitative and qualitative study**

**The two studies feel unconnected at the moment, and I wonder if more could be done to connect the findings of the two studies. The qualitative study appears to be a more general description of communication experiences of parents rather than a more in-depth exploration of the experience of information overload. This may reflect the low prevalence of information sample in the questionnaire study from which the qualitative sample was drawn. Purposive sampling to specifically invite questionnaire participants with higher CIO scores to the interview study may have enabled this in-depth exploration and I think this is a potentially limitation of the paper as it is presented.**

**Authors’ Response:** Thank you for your interesting observation. We did not do purposive sampling. It may have been interesting to speak with those with higher information overload however, we did not want to introduce selection bias but rather gain a more general understanding among our patient population.

**Below are a few additional points that I hope you might find helpful. I found that in places the manuscript was not logically organised (e.g. description of all the variables used in the quant study).**

**Introduction**

**Acknowledging the comment that there is little specific research that has been done to measure information burden in parents whose child has under gone elective surgery, I wonder if there may still be benefit to adding more contextual research that could give the reader some ideas about what factors might be influential here. E.g. Potential parental demographic, psychological variables, child medical details, HCP related factors. They are hinted at but not referenced e.g. line 99 and 100 or referenced in the discussion, line 397 Khaleel reference about association of SES with information overload.**

**Authors’ Response:** We respectfully feel that these points are adequately outlined in the introduction and discussed where relevant to our findings in the Discussion.

**Methods**

**Line 180 recommend use of “variables” rather than “factors” as it is confusing to refer to factors straight after mentioning EFA.**

**Authors’ Response:** Thank you, we have made the suggested change.

**It would add to clarity if there was a specific participants/ procedure section and separate measures section. Currently reference to some of the measures only appear in the analyses/ results e.g. prior experience of health care and language spoken at home – with the abbreviation LOTE referenced without introduction as far as I can see. A copy of the questionnaire could be added as a supplementary document for additional information.**

**Authors’ Response:** We have created a third supplement (Supplement 3) containing a full list of the questions used in the day-of-surgery survey.

**Results:**

**Regression analysis – description feels out of order by starting with reference to the interaction results – clarity / logical order would be improved if started with reference to the unadjusted results of all the variables (table I think is in supplementary) and go from there, building up to the interaction results. I haven't unfortunately had time to consider the statistical analysis in detail so I have answered "Don't know" to the question related that asks me to assess whether the statistical analysis has been completed appropriately and rigorously.**

**Authors’ Response:** In statistical modelling, the unadjusted estimates can be misleading and in fact be entirely incorrect. Adjusted estimates allow us to isolate the effect of the key variable(s) of interest after adjusting for effects of confounding variables. Given the comments from yourself and Reviewer 1, we have included a table of the unadjusted results as a table in Supplement 2. However, we disagree that the unadjusted single-variable regression estimates should be reported before the adjusted estimates in the main text, given the caveats that unadjusted estimates can be misleading. We have added a sentence referencing that the unadjusted estimates are available in Supplement 2 to the results, but we disagree about altering the order in which the results are presented given these concerns.

The following sentence has been added to the regression analysis results:

*Unadjusted regression estimates from single-variable models for total score on each candidate independent variable are reported in S2 Table 1. However, these do not reflect the true effect of each variable as this analysis does not account for the effect of confounding variables.*

**Title of table 6 could be revised for clarity – I would remove reference to stage 3 and add something that tells the reader explicitly what the barriers and enablers specifically relate to e.g. general communication or avoidance of information overload?**

**Authors’ Response:** The term stage 3 has been removed as suggested. We have adjusted the title to now read “The key themes, barriers and enablers surrounding the information received by parents regarding their child’s surgery, identified from thematic analysis of semi-structured interview transcripts “. The column headings have been revised to provide a more specific definition of the content addressed in each row.

**It would be great to see themes evidenced with quotes alongside in the table**

**Authors’ Response:**  We believe that this information will overload the table and distract from the summarised results. We have included quotes alongside the themes in the text.

**I think some of the sub codes could be revised for greater conceptual clarity:**

**e.g. Own Research or Experience - seems to relate to two different things: 1) conflict/ confusion arising from parents finding information from the internet which is different from what they have been told by hospital staff and 2) problems related to individual characteristics of parents e.g. ADHD.**

**e.g. Barriers: poor communication- appears to give a description of HCP communication that was perceived to be ineffective whereas when it comes to Enablers: “good” HCP communication is sub-divided to into different categories e.g. written, verbal, rapport.**

**Authors’ Response:** We thank the reviewer for their comments. The first subcode mentioned is referring to the notion that parents with ADHD or other health conditions found it even more confusing when their own, personal internet searches conflicted with the hospital information. We have revised the subcode to “own research and prior experiences” to add clarity. We have also separated the comment for clarity. The second example regarding poor communication reflects any form of poor communication, alongside other barriers to understanding the information given to parents (conflicting information, hospital environment and own research and prior experience). We do not feel that this code needs to be further broken down. In subsequent codes we discuss parents preferred method of communication, which we feel adds the most value to the study.

**Discussion**

**In this section it would be great to see a more detailed interpretation/ assessment of the validity, significance, and implications of the findings. For example, do the authors think that the adapted CIO could be improved upon, could/ should be used going forward? Was the low level of information overload in the sample an expected or unexpected finding? What future work could follow these studies? For example, what about the experiences of parents whose children have emergency surgery?**

**Authors’ Response:** We have amended the final paragraph in light of your suggestions.
