# Supplementary material for: Information overload and parental perspectives on information provided to parents/carers of paediatric patients undergoing elective surgical procedures
Source: PLoS One. 2024 Oct 22;19(10):e0309485. doi: 10.1371/journal.pone.0309485 (PMC11495572; doi:10.1371/journal.pone.0309485)
Supplement: Attachment — Submitted filename: Response to Reviewers .docx [file pone.0309485.s008.docx]

Below are our responses to the points raised by reviewers.

**In the discussion, detailed results (estimates and p-values) are reported on the estimated impact of education on IOC among those not identifying as Aboriginal or Torres Strait Islander. While these figures are reported in a table in the results, the authors may choose to rather highlight them under results and give a more general description in the discussion.**

**Response**: We have revised the manuscript as suggested.

**In the first paragraph of the discussion there is a referral to figure 1. This can be deleted.**

**Response**: This has been deleted.

**In the discussion, while results on education are in line with those reported in the scoping review by Khaleel et al. (2020), some are not (socio-economic factors). Is it possible to elucidate any reasons as to why?**

**Response**: The scoping review by Khaleel et al. reported that low education level and low socioeconomic status were associated with information overload in 3 and 2 studies, respectively. We have added to the Discussion section to expand on this.

**In S2 table 1 some p-values are reported as 0. These should be reported with more accuracy, e.g as <0.001.**

**Response**: The suggested change has been made to Supplement 2 Table 1.

**I have two minor suggestions regarding lines 209 and 212: the word "predict" should be changed to the past tense "predicted."**

**Response**: This change has been made as suggested in the revised manuscript.
